# Absolute or relative effects? Arm‐based synthesis of trial data

**DOI:** 10.1002/jrsm.1184

**Published:** 2015-10-13

**Authors:** S. Dias, A. E. Ades

**Affiliations:** ^1^ School of Social and Community MedicineUniversity of BristolUSA

We congratulate Hwanhee Hong and colleagues on another fascinating paper (Hong *et al.,* 2015a) arguing the case for arm‐based models for meta‐analysis.

The standard approach to meta‐analysis is the *contrast‐based* model where the information that is pooled over trials is the information of the trial‐specific *relative* treatment effect, expressed for example as a log relative risk, log odds ratio, or as a mean treatment difference. In an *arm‐based* model, it is the *absolute* log risk, log odds, or mean outcome on each arm that are pooled.

There is no doubt that arm‐based models are an intriguing alternative to the accepted understanding of meta‐analysis, and that they provide a very elegant alternative approach to network meta‐analysis (NMA). However, readers of *Research Synthesis Methods (RSM)* will have no difficulty in recognising that arm‐based models represent a radical – even revolutionary – departure from current meta‐analytic practice.

In this commentary, we begin by outlining the key differences between arm‐ and contrast‐based meta‐analysis to make clear the true extent of the implications of the claims being made. We then argue that contrast based models are to be preferred on both theoretical and practical grounds.

Hong and colleagues present a number of arguments about the core assumptions of contrast‐based models, both in their *RSM* paper (Hong *et al.,* 2015a) and previously (Zhang *et al.,* 2014, Ohlssen *et al.,* 2014, Zhang *et al.,* 2015, Hong *et al.,* 2015b), which we believe are mistaken. We offer some counter‐arguments and comment on the simulation study presented by Hong *et al.* (2015a).

## 1. Classic contrast‐based, contrast‐based plus baseline and arm‐based models

Hong *et al.* (2015a) contrast three kinds of models. We will call these (a) the “Classic Contrast‐Based” (Classic CB) model, which corresponds to the standard model for pair‐wise meta‐analysis (Higgins and Green, 2008), extended to NMA (Higgins and Green, 2008, Dias *et al.,* 2013a, Lu and Ades, 2004, Cooper *et al.,* 2009, Lu and Ades, 2006, Caldwell *et al.,* 2005), (b) “Contrast‐Based plus Baseline” (CB + Baseline), and (c) “Arm Based” (AB). It is important to be clear about notation and terminology, so we start with pair‐wise meta‐analysis and then turn to NMA.

With the contrast‐based models, particularly those used in a frequentist framework, the data to be pooled are the relative effect measures, and in its most common implementation the absolute effects are not even available to estimate. Indexing trials by *i*, the data *D*_*i*,*XY*_ are the observed relative effect measures comparing treatment *Y* to *X*, *δ*_*i*,*XY*_, which in a Bayesian setting are the “shrunken” trial‐specific estimates, and *d*_*XY*_ are the mean relative effects. Thus, the “Classic CB” model for pair‐wise (two‐treatment) data is stated as follows:

(1) $$\begin{array}{l} D_{i,XY}\sim N\left(\delta_{i,XY},V_{i,XY}\right)\;"\text{Classic}\;CB"\;\text{model}\;\text{with difference}-\text{based likelihood}\\ \delta_{i,XY}\sim N\left(d_{XY},\sigma_{XY}^{2}\right)\text{.} \end{array}$$

Arm‐based *likelihoods* (not to be confused with arm‐based models) have also been proposed (Prevost *et al.,* 2000, Smith *et al.,* 1995) particularly in a Bayesian context, partly because they avoid normal approximations for count data. This obliges us to introduce the parameter *μ*_*i*,*X*_ representing the absolute effect of the control arm X, on the appropriate scale (log risk, log odds, etc). The scale is determined by the link function *G* which transforms the relative effect parameter of interest, Θ_*i*,*XY*_ (for example a probability or rate), onto a scale where its effects can be assumed to be additive:

(2) $$G\left(\Theta_{i,XY}\right)=\alpha_{i,XY}=\mu_{iX}+\delta_{iXY}\;CB\;\mathrm{mod}el\;\text{with arm}‐\text{based likelihood}\text{.}$$

This form of contrast based model has always come in two flavours (Prevost *et al.,* 2000). The one that corresponds exactly to the “Classic CB” model (1) treats the *μ*_*i*,*X*_ as unrelated nuisance parameters. This is the approach adopted in the NMA models developed in Bristol (Dias *et al.,* 2013a, Lu and Ades, 2004, Prevost *et al.,* 2000).

The alternative is to put another hierarchical model on the trial‐specific control‐arms: $\mu_{i,X}\sim N\left(m_{X},\sigma_{m}^{2}\right)$ (Achana *et al.,* 2013, Dias *et al.,* 2013c). This is the “CB + baseline model”, or alternatively a bivariate normal model on the *μ*_*i*,*X*_ and the *δ*_*i*,*XY*_ could be adopted (van Houwelingen *et al.,* 1993).

To see how this idea works in NMA, we need to complicate the notation a little. First, we will consider trial‐specific treatment indices *k* = 1, 2, 3 …, and we distinguish between the relative effect of the *k*‐th treatment relative to the control treatment on that trial $\delta_{i,t_{1}t_{k}}$, and its effect relative to the reference treatment 1, $\delta_{i,1t_{k}}$, which may or may not be present in trial *i*. With this extended notation, the “Classic CB” model makes the trial‐specific control arms arbitrary and unrelated.

(3) $$G\left(\Theta_{i,t_{k}}\right)=\alpha_{i,t_{k}}=\mu_{it_{1}}+\delta_{i,t_{1}t_{k}},\;\mu_{it_{1}}\sim N\left(0,100^{2}\right)\;\text{Classic}\;CB\;\text{model}\;for\;NMA$$

Note that often, subscript *t*_1_ is dropped from $\mu_{it_{1}}$, as it is redundant. Clearly, there is no way of estimating an absolute effect for any treatment from the “Classic CB” model.

We note in passing that in another paper Hong *et al.* (2015b) claim that the assumption of exchangeability of “effects relative to an arbitrary and nonconstant baseline remains much to assume”. This misses the point that exchangeability of the effects *δ*_*i*,1*k*_ relative to treatment 1 *implies* exchangeability of *all* the *δ*_*i*,*jk*_ (Lu and Ades, 2009).

In the “CB + baseline” model, a model is placed on the absolute effect of reference treatment 1 in trial *i*. Note that it is not put on the trial control arms, as these are not necessarily the same treatment—care should be taken that only the effect of treatment 1 is modelled. This allows absolute effects on the chosen scale (for example the log‐odds or log), *a_k_*, to be calculated for any treatment based on the included trial data. The model can be written as

(4) $$\begin{array}{l} G\left(\Theta_{i,t_{k}}\right)=\alpha_{i,t_{k}}=\mu_{i1}+\delta_{i,t_{1}t_{k}},\;\mu_{i1}\sim N\left(m,\sigma_{m}^{2}\right)\;CB+\text{baseline}\;\text{model}\;for\;NMA\\ a_{k}=m+d_{1t_{k}}\text{.} \end{array}$$

Note that in the “CB + baseline” model, information on the baseline will back‐propagate onto the relative effect parameters. It is this property of “CB + Baseline” models that makes the relative effect estimates vulnerable to misspecification of the absolute effects.

We can now set out the arm‐based models proposed by Hong *et al.* (2015a) as

(5) $$G\left(\Theta_{i,t_{k}}\right)=\alpha_{i,t_{k}}\sim N\left(a_{t_{k}},\sigma_{t_{k}}^{2}\right)\;\text{AB}\;\text{model}\;for\;NMA\text{.}$$

The first feature we notice is that there are no relative treatment effects in equation (5). Mean “treatment effects” on the linear predictor scale are manufactured from the treatment‐specific means as *d*_*XY*_ = *a*_*Y*_ − *a*_*X*_. Note that while the CB modeller working on binary data is committed to specify whether effects are additive on a continuous, log, or log‐odds scale, the AB modeller is free to report risk differences, *G* ^− 1^(*a*_*Y*_) − *G* ^− 1^(*a*_*X*_), relative risks *G* ^− 1^(*a*_*Y*_)/*G* ^− 1^(*a*_*X*_), or odds ratios (*G* ^− 1^(*a*_*Y*_)*G* ^− 1^(1 − *a*_*X*_))/(*G* ^− 1^(*a*_*X*_)*G* ^− 1^(1 − *a*_*Y*_)). For binary outcomes, both probit and logit link functions have been proposed in arm‐based models, presumably giving virtually identical results. This “model‐free” approach to relative effect estimation has the advantage that it makes no assumptions, but, as we see below, it comes with a heavy price in posterior variance of the relative effects.

In the latest paper in RSM Hong *et al.* (2015a) extend AB modelling to multiple outcomes, and run simulations that compare “Classic CB”, “CB + baseline”, and “AB” models in various styles. In previous work developing AB models Zhang *et al.* (2014) have criticised the “Classic CB” model on the grounds that, because it contains no model for absolute effects, it cannot report overall event rates without making further assumptions (although some would see this as an advantage of “Classic CB”). Other papers have examined the concept of inconsistency in NMA (Zhang *et al.,* 2015) and looked at the role of AB models in drug development (Ohlssen *et al.,* 2014).

### 1.1. Comparing arm‐based and contrast based models

- *Fundamentals of meta‐analysis*

Although much of the arm‐based literature casts the debate as specifically relevant to NMA, it should be clear that the central claim being made is equally relevant to pair‐wise (i.e. two‐treatment) meta‐analysis (MA). The proposal that one should pool absolute arm effects rather than relative treatment effects goes against the entire tradition of MA, in which the absolute event rates have been viewed as nuisance parameters, and as highly variable, while the relative effects, expressed on a suitable scale, have been seen as relatively stable. One might go further, and say that arm‐based models run against the entire tradition of epidemiological statistics in which relative effects – for example of risk factors – are pooled in multiple 2‐by‐2 tables (Mantel and Haenszel, 1959, Zelen, 1971).

We also believe that arm‐based pooling effectively breaks randomisation, and in fact runs against the entire way in which randomised controlled trials are designed, analysed, and used.

While “AB” models take an extreme position, many of us have also argued against “CB + baseline” models, because, unless the baseline model is correctly specified, the relative effect estimates will be biased. For example, one often finds that outcomes on the control arm have improved over time, because less severe populations are entered into more recent trials while the relative effect has stayed constant. If one random effects model is put on the baselines and another on the relative effects, and both are estimated at the same time, we expect the baseline effects in earlier studies to be shrunk upwards, and relative effects under‐estimated, while the relative effects in more recent studies will be over‐estimated. Senn (2010) has argued forcefully against “CB + baseline” models for these reasons. “AB” models will be even more likely to produce biased treatment effects, if one accepts the commonly made assumption that it is the relative effects which are exchangeable across trials.

*Evidence synthesis for decision‐making*

Working as we do on evidence synthesis in the decision making context, we are well aware the absolute event rates often play a more important role than the relative treatment effects when treatment recommendations are made. Thus, we fully accept that there needs to be a model for the absolute effect that one would see on at least one of the treatments, from which absolute effects can be recovered for all treatments.

Where we differ from proponents of AB models is that we believe that, while randomised controlled trials are unquestionably the best data sources to inform relative effects, the data sources that best inform the absolute effects might be cohort studies, a carefully selected subset of the trials included in the meta‐analysis, or expert opinion. Manufacturers making submissions on new products to the National Institute for Health and Care Excellence (NICE) in the UK are asked to explain the basis on which they have chosen the evidence for absolute effects, and this is quite distinct from trial evidence from which relative effects are sourced (National Institute for Health and Clinical Excellence, 2012).

The disadvantage of an arm based model is that it *obliges* the modeller to use exactly the same data sources for both absolute and relative effects, whereas the natural way to carry out health technology assessments of alternative treatments is to apply a “transportable” relative effect estimate to an independently estimated baseline model, which can be carefully tailored to represent the relevant target population. This is what is recommended in standard texts (Hunink *et al.,* 2001, Drummond *et al.,* 1997, National Institute for Health and Clinical Excellence, 2012).

One might add that in many cases the entire debate about whether a treatment should be adopted hinges on the “threshold” baseline risk at which the treatment – whose relative effect is assumed to remain constant in ratio terms – becomes cost effective, or at which the clinical benefits outweigh the side‐effects. Statin treatment is perhaps the prime example (Stone *et al.,* 2013, National Clinical Guideline Centre, 2014). It is not clear how an arm‐based approach could be modified or extended to produce the kind of analysis that is needed to support decision makers in this context, or indeed in any context.

*The empirical question*

We are not sure whether proponents of “AB” models would agree, but to some extent the choice between arm‐ and contrast based models could be regarded as an empirical question: do we find in practice that relative effects, given that an appropriate scale has been chosen, are more stable than absolute effects? This is the kind of question that has been studied previously using L'Abbé plots (L'Abbe *et al.,* 1987, Song, 1999).

In the example presented by Hong *et al.* (2015a), the between study variances in “AB” models were considerably greater than in the “CB” models. It is important to note that the posterior precision of the mean relative effects *d*_1*k*_ was quite severely degraded as a result, as can be seen from the wide credible intervals of AB models in Fig. 6. Of course, poor posterior precision is not necessarily inappropriate posterior precision, but the effect of the arm‐based model is to allow the huge variation in absolute effects to affect the estimates of relative effects, while contrast‐based pooling is precisely designed to insulate them from this. Global Goodness of fit and Deviance Information Criteria do not provide a good basis for model choice as different patterns of shrinkage can be expected. The choice should be made on principled and empirical grounds, with a view to minimising bias, not maximising goodness of fit or parsimony.

### 1.2. Missing‐ness of treatment arms in NMA

The key assumption in all the CB models is the exchangeability of the trial‐specific treatment effects *δ*_*i*,*XY*_ across the entire ensemble of trials. Specifically this means that the *δ*_*i*,*XY*_ in XY trials are exchangeable with *δ*_*i*,*XY*_ in XZ and YZ trials and indeed in all the trials, including those that have neither X nor Y arms. In previous papers (Caldwell *et al.,* 2005, Dias *et al.,* 2011) we have said that this was equivalent to the arms being “missing at random” (MAR). By this we meant that missing‐ness was unrelated to any factors that influenced the relative treatment effects *δ*_*i*,*XY*_. Hong *et al.* (2015a) claim that CB models require “missing completely at random” (MCAR) and will not work with MAR, because MAR would rule out missing‐ness being related to *absolute* efficacy. Of course, if “Classic CB” models for NMA were vulnerable to missing‐ness that was related to absolute effects, this would be a most serious criticism because this type of missing‐ness is surely a very common occurrence. But “Classic CB” models are quite obviously *not* vulnerable to this kind of missing‐ness, because it does not imply that missing‐ness depends on relative effects. The *only* requirement for CB models is that missing‐ness is unrelated to the relative effects, which is related to the requirement that the trial‐specific relative effects are exchangeable (Lu and Ades, 2009).

Hong *et al.* (2015a) correctly note that CB models are vulnerable to relationships between relative treatment effects and baseline severity. In a CB framework this – as with any relative effect modification – is typically handled by a meta‐regression of the trial‐specific relative effects against absolute response to the reference treatment, but *without* putting a model on the reference treatment (Dias *et al.,* 2013b). What is less clear is how AB modellers would cope with this pattern in the data. Covariates can readily be added to AB models, but there appears to be no way of adding a covariate to a *relative* effect, or putting different covariates on relative and absolute effects.

## 2. Simulation studies

The simulation studies reported in Hong *et al.* (2015a) relate to an extension of AB models to multiple outcomes, and they compare certain variants of AB, and “CB + baseline” models, with “Classic CB” (labelled “LAREhom” in Hong *et al.* (2015a), for Lu & Ades Random Effects homogeneous variance). The purpose is to compare the three models in three‐treatment networks under different forms of missing‐ness: MCAR (completely at random), MAR, and MNAR (not at random), and with different degrees of missing‐ness, approximately 10%, 20%, and 40%. The target outputs of the simulations are (a) the arm means *a*_1_, *a*_2_, *a*_3_ and (b) the mean relative effects *d*_12,_ *d*_13_ (an identity link is used so we ignore the back transformation *G* ^−*1*^).

Readers should immediately be asking themselves, how can the “Classic CB” model, which does not concern itself at all with absolute effects be made to generate estimates of the absolute effects *a*_1_, *a*_2_, *a*_3_?! It is at this point that we must confess that in a number of publications we have, *to our eternal regret*, created an estimate of *a*_1_ by averaging the absolute event rates on the reference treatment 1. We have done this simply to illustrate the process by which absolute effects *a*_*k*_ can be composed from the relative effects *d*_1*k*_ and an estimate of the absolute effect on the reference treatment *that does not originate from the NMA itself*. In spite of this tactical blunder, it should be clear, and we believe it *is* clear from our specification of the “Classic CB” models in previous publications (Dias *et al.,* 2013c, Lu and Ades, 2004), that these estimates of *a*_1_ have nothing to do with the “Classic CB” relative effects model and they have the effect of allowing us to estimate absolute effects without taking the risk of introducing bias into the relative effects. We are therefore quite surprised that Hong *et al.* (2015a) claim that their simulations show that “Classic CB” “fails to estimate the *a*_*k*_ correctly”. The truth is that “Classic CB” *has nothing whatsoever to say* about absolute effects at all, and this is perfectly obvious from our publications.

Needless to say, “AB” and “CB + baseline” models both perform well in terms of bias, MSE, and coverage, on *a*_*k*_ in simulations, but, as we explain above, the estimates of *a*_*k*_ obtained by these models are unlikely to be of practical value, unless they happen to best represent the target population. Moreover, it is highly likely that they will be accompanied by biased estimates of the relative effects *d*_1*k*_. In the case of “AB” models the estimates of *a*_*k*_ are, in our view, completely arbitrary and unusable. They have an interpretation that makes about as much sense as the average distance between carrots and Cadillacs.

When it comes to the *d*_1*k*_, the simulation studies show that “Classic CB” performs as well as “CB + baseline” model in MCAR scenarios, and better than “CB + baseline” model for “MAR” and MNAR scenarios. (We put the MAR in quotes as they are generated from AB models, and we are uncertain as to whether they would be MAR with respect to relative effects). The main problems for “Classic CB” are the “MAR” scenarios. It is important to appreciate that *all* the missing‐ness scenarios have the missing‐ness of treatments depending on a function of the simulated *absolute* effects on one or *both* of the outcomes. For example in “MAR” the presence of treatment 1 depends on the sum over the two outcomes of the absolute effects on treatment 2, while the presence of treatment 3 depends on the difference between outcomes on treatment 2. It is not immediately clear what this implies about missing‐ness with respect to relative effects. It is therefore difficult to interpret the results, in which “Classic CB” performs worse as the degree of missing‐ness increases, because the data are generated *with* correlations while the “Classic CB” models used to analyse each simulated dataset assume zero correlations. We note that “Classic CB” models for multiple outcomes which incorporate correlations have been proposed (Efthimiou *et al.,* 2014, Bujkiewicz *et al.,* 2014, Bujkiewicz *et al.,* 2013, Schmid *et al.,* 2014) and they would perhaps provide a fairer comparison.

Regarding the MNAR scenarios, Hong *et al.* (2015a) note that “Classic CB” produces “oddly unbiased” estimates of the *d*_1*k*_; “oddly” because both *a*_1_ and *a*_*k*_ are biased, but “to the same degree”. This is, in fact, *exactly* what would be expected. A great many MNAR scenarios were tested, in which missing‐ness was an extremely complex function of simulated absolute effects on different treatments and outcomes, but none clearly implying a failure of MAR on relative effects. We take these results as a vindication of the arguments set out above to the effect that placing a model on baseline effects makes relative effects vulnerable to misspecification of the baseline model.

## 3. Conclusions

We believe that AB models are thoroughly misguided, and a huge step back from the separation of absolute and relative effects that has been at the core of modern epidemiology and biostatistics for so long. The use of AB models would risk seriously biased estimates of relative treatment effects, with over‐inflated posterior variance, and block the ability to “transport” relative effects into new absolute effect scenarios, a basic principle in health technology assessment.

Absolute effects should be estimated independently, and the appropriate evidence sources are most likely *not* to be trials, let alone the same trials which inform the relative effects. The exchangeability assumptions on which “Classic CB” models are based imply MAR with respect to relative effects, and we remain unconvinced by the simulation studies so far presented.

The presentations of AB models in *RSM* and other journals are superb examples of Bayesian data analysis, and while we disagree profoundly with their assumptions, the authors have succeeded in making us reflect more precisely on the assumptions we are making in evidence synthesis models, and on how well these assumptions are supported by theory or empirical evidence. One particular issue that their papers raise is scale. Some of the important benefits enjoyed by CB modellers depend on an appropriate choice of link function, and it is perhaps not always recognised that choice of which treatment is best can be sensitive to this (Caldwell *et al.,* 2012, van Valkenhoef and Ades, 2013, Norton *et al.,* 2012). Hong et al.'s presentation of “scale‐free” AB models surely challenges our usually blind adoption of log‐odds, log, or even identity links, and it encourages us to pay much more careful attention to the fundamental properties of both frequency counts in different situations, and the scales of measurement used for continuous outcomes.
